# Interaction of Metallic Nanoparticles With Biomimetic Lipid Liquid Crystalline Cubic Interfaces

**DOI:** 10.3389/fbioe.2022.848687

**Published:** 2022-03-15

**Authors:** Jacopo Cardellini, Costanza Montis, Francesco Barbero, Ilaria De Santis, Lucrezia Caselli, Debora Berti

**Affiliations:** ^1^ Department of Chemistry “Ugo Schiff”, University of Florence, Florence, Italy; ^2^ CSGI, Consorzio Sistemi a Grande Interfase, University of Florence, Sesto Fiorentino, Italy; ^3^ Department of Chemistry, University of Turin, Turin, Italy; ^4^ Department of Pharmacy, University of Copenhagen, Copenhagen, Denmark

**Keywords:** gold nanoparticles, silver nanoparticles, biomimetic systems, nano-bio interface, lipid liquid crystals, cubosomes, nano-bio interactions, cubic membranes

## Abstract

In the past decades, events occurring at the nano-bio interface (i.e., where engineered nanoparticles (NPs) meet biological interfaces such as biomembranes) have been intensively investigated, to address the cytotoxicity of nanomaterials and boost their clinical translation. In this field, lamellar synthetic model membranes have been instrumental to disentangle non-specific interactions between NPs and planar biological interfaces. Much less is known on nano-biointeractions occurring at highly curved biological interfaces, such as cubic membranes. These non-lamellar architectures play a crucial -but far from understood-role in several biological processes and occur in cells as a defence mechanism against bacterial and viral pathologies, including coronaviruses infections. Despite its relevance, the interaction of cubic membranes with nano-sized objects (such as viral pathogens, biological macromolecules and synthetic NPs) remains largely unexplored to date. Here, we address the interaction of model lipid cubic phase membranes with two prototypical classes of NPs for Nanomedicine, i.e., gold (AuNPs) and silver NPs (AgNPs). To this purpose, we challenged lipid cubic phase membranes, either in the form of dispersed nanoparticles (i.e., cubosomes) or solid-supported layers of nanometric thickness, with citrate-stabilized AuNPs and AgNPs and monitored the interaction combining bulk techniques (UV-visible spectroscopy, Light and Synchrotron Small-Angle X-ray Scattering) with surface methods (Quartz Crystal Microbalance and Confocal Laser Scanning Microscopy). We show that the composition of the metal core of NPs (i.e., Au vs Ag) modulates their adsorption and self-assembly at cubic interfaces, leading to an extensive membrane-induced clustering of AuNPs, while only to a mild adsorption of isolated AgNPs. Such differences mirror opposite effects at the membrane level, where AuNPs induce lipid extraction followed by a fast disruption of the cubic assembly, while AgNPs do not affect the membrane morphology. Finally, we propose an interaction mechanism accounting for the different behaviour of AuNPs and AgNPs at the cubic interface, highlighting a prominent role of NPs’ composition and surface chemistry in the overall interaction mechanism.

## 1 Introduction

During the last decades, inorganic nanoparticles (NPs) have been extensively investigated as possible building blocks for the development of therapeutic/diagnostic systems for biomedical applications: from the diagnosis and treatment of pathologies, to the recovery of damaged tissues, to the targeted, specific delivery of drugs, to the theranostics of serious diseases such as cancer, bacterial and viral infections, (Salvioni et al., 2017; Anees Ahmad et al., 2020; Yaqoob et al., 2020), the designed nanomedicine applications of inorganic NPs and, in particular, of metallic NPs, are countless (Alkilany et al., 2013; Muthu et al., 2014; Lombardo et al., 2019).

Despite this extremely promising potential, the clinical translation of NPs is still very limited, also due to a lack of comprehension and control of the NPs biological fate once in living organisms (Nel et al., 2009). Understanding the energetic contributions that rule interactions at the nano-biointerface (i.e. where NPs meet biological barriers, specifically cell membranes), is very challenging, due to the high compositional heterogeneity of biomembranes and the intrinsic variability of the biological environment. In this context, model membranes of lamellar nature (i.e., composed of a flat lipid bilayer (Montis et al., 2014a)) have proved to be instrumental to unravel non-specific interactions of nanomaterials with lipid interfaces and relate their behavior with the therapeutic efficacy and/or toxicity of NPs in biological organisms (Hélix-Nielsen, 2018; Pfeiffer et al., 2019; Montis et al., 2020a).

Conversely, the interaction of NPs with more complex membranous architectures, characterized by a non-lamellar nature, is practically unexplored. The so-called “cubic membranes”, for instance, are biologically relevant structures characterized by a high membrane curvature and translational order (Almsherqi et al., 2006). Their 3D structure possess a cubic symmetry and consists of bicontinuous aqueous non-intersecting nanochannels separated by a lipid bilayer (Mezzenga et al., 2019). Several reports demonstrated the permanent or transient-occurrence of such a non-lamellar phase in cell membranes under starvation, viral infection, oxidative stress and other pathological conditions (Deng and Mieczkowski, 1998; Almsherqi et al., 2009; Mezzenga et al., 2019). Importantly, it has been found that coronavirus infections (e.g. SARS-CoV-2, SARS-CoV, or MERS-CoV) are connected with the formation of cubic membranes in host cells (Knoops et al., 2008; Zhang et al., 2020; Deng and Angelova, 2021), which has been proposed as an evolutionary defence mechanism. Moreover, this membrane organization plays crucial roles in several biological processes, such as membrane fusion and fission.

On the synthetic side, these natural highly curved biological membranes inspired the development of artificial lipid-based architectures of cubic geometry, currently used in several technological fields, ranging from protein crystallization (Cherezov et al., 2002; Meikle et al., 2017), to nanomedicine (Yaghmur and Mu, 2021) and nutritional science (Yaghmur, 2019).

Synthetic cubic lipid systems can be formulated as “cubosomes” (Gustafsson et al., 1996), i.e. water-dispersed nanosized particles with internal cubic structure. The colloidal stability of such particles in water is traditionally provided by amphiphilic copolymers adsorbed onto their surface (Chong et al., 2015), even though novel classes of stabilizer-free cubosomes emerged more recently (Zabara et al., 2019). Cubosomes have an enormous potential as vectors for *in vivo* targeted delivery of drugs, antimicrobials and active principles (Barriga et al., 2019; Zabara et al., 2019), due to the extraordinarily high encapsulation efficiency of both hydrophilic and hydrophobic molecules, compared to more traditional liposomal carriers. The formulation of hybrid inorganic NPs/cubosome systems can further extend their potential in nanomedicine, providing the lipid matrix with stimuli-responsive features. In this respect, understanding the interactions of inorganic NPs with the lipid interface of cubosomes has proved instrumental for the successful design of smart hybrid carriers for magnetically guided delivery and controlled release of drugs (Montis et al., 2015; Mendozza et al., 2018; Caselli et al., 2021a).

Beside this promising applicative potential, investigating the interaction of NPs with cubosomes offers a fresh perspective on nano-biointeractions, shifting the focus to the interface of cubic biomembranes. In this respect, artificial cubic phase systems, such as solid-supported cubic phase films, have been proposed as possible biomimetic platforms only recently (Dabkowska et al., 2017a; Dabkowska et al., 2017b; Caselli et al., 2022). These synthetic mimics aim at shedding some light on the mysterious role of cubic membranes in Nature, as well as investigating their response to nano-sized objects, from artificial nanomaterials to biological NPs, e.g. virus and biogenic vesicles. As an example, in a recent work we demonstrated that synthetic cubic phase membranes are significantly more resistant than lamellar ones against the destructive effect of positively charged AuNPs with different morphologies (Caselli et al., 2022). This effect was connected to the different geometry of the membrane, able to drive profound differences in the interactions established at the nano-bio interface.

However, except for few pioneering studies in the field, the interaction of such cubic interfaces with NPs still represents an almost unexplored research field.

In the present work, we studied the interaction of prototypical metallic NPs for medical applications (i.e. Ag- and AuNPs) with stabilizer-free cubosomes, synthetized according to recent preparation protocols (Zabara et al., 2019). We employed cubosomes either dispersed in water or adsorbed onto a solid support. Such artificial lipid models allowed us to study the effect of the core NPs composition (i.e. Ag- vs AuNPs) on cubic phase membranes, under simplified and controlled conditions. From the membrane side, the absence of a steric stabilizer on cubosomes enabled to investigate the interaction of NPs with a “naked” cubic interface, not mediated by a polymeric layer, in more similar conditions as the ones in living cells. From the NPs side, citrated Ag- and AuNPs were selected. Citrate anions are weakly associated to the metallic surface and are easily displaced by biomolecules commonly present in living organisms, such as serum proteins and membrane lipids (Perera et al., 2018; Wang et al., 2019; Montis et al., 2020b). Thus, the selected particles allow a direct comparison of the interaction of two different metallic surfaces with the cubic lipid interface, minimizing the mediation of the coating agent. With this aim, we monitored the interaction of Ag- and AuNPs with cubosomes through a combination of bulk techniques (e.g. UV-vis spectroscopy (UV-vis) and Small Angle X-ray Scattering (SAXS)) and surface methods, i.e. Quartz Crystal Microbalance (QCM) and Confocal Laser Scanning Microscopy (CLSM). Overall, we show how the interaction crucially depends on the composition of the metallic NPs, leading to weaker interactions with the AgNPs and to irreversible clustering of AuNPs on the cubic phase membrane. These findings broaden our current knowledge of the chemical-physical parameters which govern the interaction at the nano-biointerface, extending the investigation to cubic interfaces of high biological relevance.

## 2 Materials and Methods

### 2.1 Materials

Tetrachloroauric (III) acid, silver (I) nitrate, trisodium citrate dihydrate, Glycerol monooleate (GMO), and tannic acid were provided by Sigma-Aldrich (St. Louis, MO). 1,2-dioleoyl-sn-glycero-3-phosphocholine (DOPC) and 1,2-dioleoyl-3-trimethylammonium-propane (DOTAP) were provided by Avanti Polar Lipids, as well as 18:1 Cyanine 5 Phosphatidylethanolamine hydrophobic dye. The lipid dye β-Bodipy TM FL C12-HPLC were provided by Thermofisher. All chemicals were used as received. Milli-Q grade water was used in all preparations (conductivity: 0.056 μS/cm, resistivity: 18.2 MΩ cm at room temperature).

### 2.2 Synthesis of AuNPs

Anionic gold nanospheres were synthesized according to a kinetically controlled seeded growth method (Bastús et al., 2011).

#### 2.2.1 Synthesis of Au seeds

Briefly, 250 ml of a 2.2 mM sodium citrate aqueous solution was brought to boiling temperature under constant and vigorous magnetic stirring in a three-necked flask. When the solution reached the boiling temperature, 1 ml of HAuCl_4_ was injected. The solution was further boiled for 10 min until it acquired a light pink colour. The resulting seeds are citrate coated particles of about 10 nm.

#### 2.2.2 Growth of AuNPs

After the seed formation, 25 ml of seed dispersion were extracted, and 25 ml of sodium citrate 2.2 mM were injected. The reaction was cooled until 85–90°C. 1 ml of HAuCl_4_ 25 mM was added in the flask. The colour of the solution turns red, and the resulting particle size is about 21 nm. The growth process was repeated twice to obtain AuNPs with the required size.

### 2.3 Synthesis of AgNPs

Anionic silver nanospheres were synthesized according to a kinetically controlled seeded growth method (Bastús et al., 2014).

#### 2.3.1 Synthesis of Ag seeds

As for Au nanospheres, 100 ml of a 5 mM sodium citrate aqueous solution was brought to boiling temperature under constant and vigorous magnetic stirring in a three-necked flask. Then 200 μL of tannic acid 2,5 mM and 1 ml of AgNO_3_ were injected sequentially (delay time 3 min). After 10 min, 25 ml of Ag seeds were extracted, and 25 ml of sodium citrate 5 mM were added.

#### 2.3.2 Growth of AgNPs

After the seed formation, the reaction mixture was cooled until 90°C. Then, 250 μL of tannic acid (2,5 mM) and 250 μL of AgNO_3_ (5 mM) were sequentially injected (time delay ∼ 1 min). The solution was heated for another 10 min before the particle characterization.

### 2.4 Liposomes and Cubosomes Preparation

For the preparation of DOPC (1,2-Dioleoyl-sn-glycero-3-phosphocholine)/DOTAP (Dioleoyl-3-trimethylammonium propane) (70/30 mol%) liposomes for QCM measurements, the proper amount of the two lipids were firstly dissolved in chloroform in 40 ml glass vials. For CLSM measurements, the lipid dye β-Bodipy TM FL C12-HPLC (Thermofisher), dissolved in chloroform, was added to the mixture at a 0.1% mol concentration with respect to the total lipid (DOPC + DOTAP) amount. and a lipid film was obtained by evaporating the solvent under a stream of nitrogen and overnight vacuum drying. The film was then swollen and suspended in a 100 mM NaCl water solution by vigorous vortex mixing to obtain a final 0.5 mg/ml lipid concentration. The resultant multilamellar vesicles (MVLs) in water were tip sonicated with a Digital Sonifier Model 450 (Branson, Hampton, NH, United States), provided with a Horn Tip (diameter 25.4 mm), in an intermittent-pulse mode (5 s), with a power of 400 W (amplitude 50%), for 15 min to obtain a homogeneous dispersion of unilamellar vesicles with a narrow size distribution. Glycerol monooleate (GMO)-based cubosomes were prepared according to a recent protocol (Zabara et al., 2019). A proper amount of GMO was weighted and suspended in in ultrapure milli-Q water by vigorous vortex mixing to obtain a final 4 mg/ml lipid concentration. The resultant dispersion was tip-sonicated for 30 min. For CLSM measurements, the cubosomes dispersion was added to a dry film of a lipid dye (18:1 Cyanine 5 Phosphatidylethanolamine), previously formed on the bottom of a glass vial from a methanol solution, to obtain a final dye’s concentration within cubosomes of 0.1% mol.

### 2.5 UV-Visible Spectroscopy

UV-Vis spectra were collected with a Cary 3500 UV-Vis spectrophotometer.

### 2.6 Small Angle X-Ray Scattering

Small Angle X-Ray Scattering profiles were measured on AuNPs, AgNPs and hybrid dispersions using a Xeuss 3.0HR (Xenocs) in-strument equipped with a Genix3D (Cu) X-Ray source and a Dectris 1 M Eiger detector. Samples were contained in glass capillary tubes of thickness 1.5 mm. Data from each sample were acquired at Sample-Detector (S-D) distances of 450 and 1800 mm for 300 s. Intensities were normalized with respect to transmission and sample thickness. After data reduction, the contribution of the sample holder and solvent (water) was subtracted from the sample intensity. The wave vector range (Q-range) accessed in the experiments was The data were analysed with the SasView software. The GMO cubosomes dispersion was analysed at SAXS beamline of synchrotron radiation Elettra, Trieste (Italy) operated at 2 GeV and 300 mA ring current. The experiments were carried with and SAXS signal was detected with Pilatus 3 1 M detector in q-range from 0,009 to 0,7 recording the SAXS curves in a glass capillary.

### 2.7 Cryo-TEM

3 μL of GMO-based cubosomes were applied on glow-discharged Quantifoil Cu 300 R2/2 grids and plunge frozen in liquid ethane using an FEI Vitrobot Mark IV (Thermo Fisher Scientific). Excess liquid was removed by blotting for 1 s (blot force 1) using filter paper under 100% humidity and 10°C. Cryo-EM data were collected at Florence Center for Electron Nanoscopy (FloCEN), University of Florence (Italy), on a Glacios (Thermo Fisher Scientific) at 200 kV equipped with a Falcon III detector operated in counting mode. Images were acquired using EPU software with a physical pixel size of 2,5 Å and a total electron dose of ∼50e−Å^−2^ per micrograph.

### 2.8 ζ-Potential Measurements

Zeta Potential determination were performed using a Brookhaven Instrument 90 Plus (Brookhaven, Holtsville, NY). Each measurement was an average of ten repetitions of 1 minute each and repeated ten times. Zeta potentials were obtained from the electrophoretic mobility u, according to Helmholtz-Smoluchowski equation:

$$\zeta =\frac{\eta }{\varepsilon }\times u$$ (1)

with η being the viscosity of the medium, ε the dielectric permittivity of the dispersing medium. The Zeta Potential values are reported as averages from ten measurements.

### 2.9 Quartz Crystal Microbalance

QCM experiments were performed on a Q-Sense E1 instrument (Q-Sense, Gothenburg, Sweden). The instrument was equipped with a flow liquid cells (0.5 ml internal volume), containing a quartz sensor with 4.95 MHz fundamental resonance frequency, mounted horizontally. The active surface of the sensors (∼1 cm^2^) was coated with a thin SiO_2_ layer (∼100 nm thick). Prior to use, the sensors were bath sonicated in pure acetone and ethanol, then extensively washed with Milli-Q water and dried with nitrogen flux. After that a plasma cleaner was used for 10 min to completely oxidize the surface. The experiments were performed at room temperature and solvent exchange in the measurement chamber was achieved with a peristaltic pump. First, the sensors were placed in the chambers and water was injected at a flow rate of 0.5 ml/min), the frequencies (f) were measured for the odd harmonics (1st–13th). A stable baseline for f of the different harmonics was ensured before the injection of the vesicles. The QCM curves reported are normalized by the overtone number.

### 2.10 Confocal Laser Scanning Microscopy

A Leica CLSM TCS SP8 confocal microscope, operating in inverted mode, with a 63 × 1.3 numerical aperture water immersion objective, was used to image the lipid-based surface structures in water excess. The β-Bodipy TM FL C12-HPLC (Thermofisher) lipophilic dye was used to label DOPC/DOTAP liposomes; the fluorescence of this probe was excited at 488 nm and collected in the 498–530 nm emission range with a Phomultiplier tube (PMT). The fluorescence of 18:1 Cyanine 5 Phosphatidylethanolamine (Avanti Polar Lipids) was used to label GMO cubosomes, employing an excitation wavelength of 633 nm, while the fluorescence was collected in the 650–700 nm emission range with a PMT. Images were taken with a resolution of 512 × 512 pixels using a 400 Hz bidirectional scan with each scanning line averaged four times. Leica software was used to create three-dimensional reconstructions of the z-stacks.

## 3 Results

### 3.1 Metallic NPs and Cubosomes

**FIGURE 1:**
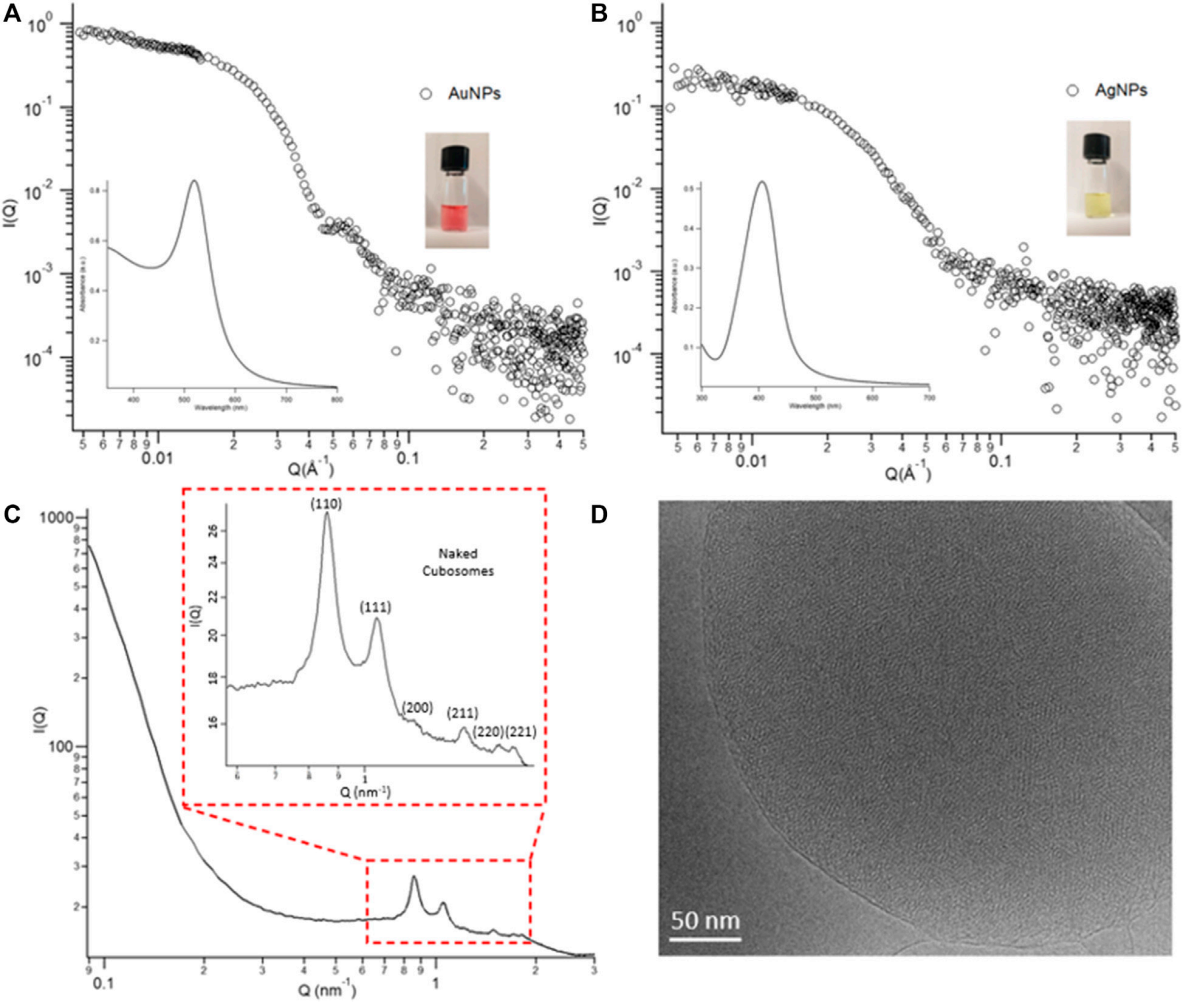
Panel **(A)** Physicochemical properties of NPs. SAXS profiles and UV-vis spectra (insets) of AuNPs and AgNPs in water (at a concentration of 8.3 × 10^−10^ M). Panel **(B)** Structural characterization of cubosomes. **(C)** SAXS profile of cubosomes in water at a GMO concentration of 4 mg/ml. The inset depicts the inner cubic Pn3m structure of cubosomes. **(D)** Representative Cryo-TEM image of the Pn3m inner architecture of cubosomes.

Metallic NPs possess unique optical properties due to Surface Plasmon Resonance. Depending on composition, size, shape, and chemical environment, nanometals exhibit different absorption in the UV-Visible spectral region, endowing their dispersions with a characteristic colour. Au and Ag nanospheres typically show size-dependent shades of red and yellow, respectively, with progressive darkening as NPs’ size increases. Here, spherical citrate-capped Au- and AgNPs were synthesized following a fast and reproducible protocol (Bastús et al., 2011; Bastús et al., 2014) consisting in the production and homogeneous growth of particles’ seeds. The concentration, size, surface properties, as well as the optical features of NPs, are summarized in Table 1 (see also S.1 of Supplementary Material for further characterization). The average size of NPs was determined with Small-Angle X-Ray Scattering (Figures 1A,B). The gyration radii of the particles were obtained through a model-free Guinier analysis of the SAXS profiles of Figures 1A,B, reported in section S.1 of Supplementary Material. Considering spherical particles, we obtained AuNPs and AgNPs of about 20 nm in size and low polydispersity (Table 1). AgNPs and AuNPs UV-Vis spectra in water are characterized by an intense absorbance centred at 406 and 519 nm respectively (see insets in Figures 1A,B), in agreement with previous reports on particles of similar size (Bastús et al., 2011; Bastús et al., 2014). The anionic capping agents of NPs (citrate molecules for AuNPs, and citrate and tannic acid (mole ratio ∼500/1) molecules for AgNPs), confer an overall negative surface charge to the particles’ surface, been determined through zeta potential measurements (Table 1).

**TABLE 1:** Physicochemical properties of NPs obtained by UV-Vis, SAXS, and ζ -potential measurements. The reported concentration values correspond to the concentration of NPs in the aqueous dispersion, obtained from the synthesis. Before each measurement, AgNPs concentration has been adjusted by dilution, to match the one of AuNPs (8.3 × 10^−10^ M).

	Conc.(M)	λ_max_ (nm)	Size (nm)	ζ-pot.(mV)
AuNPs	8.3 × 10^−10^	519	20 ± 1	−22.5 ± 1
AgNPs	1.8 × 10^−8^	406	22 ± 1	−18.9 ± 0.3

Stabilizer-free glycerol monooleate (GMO)/water cubosomes at a 4 mg/ml lipid concentration have been prepared by dispersing the GMO bulk phase in water excess, according to a recently published protocol. (Zabara et al., 2019). Figure 1C reports the structural features of their internal architecture. The multiple Bragg peaks in the scattering profile are consistent with a highly ordered lipid cubic phase with a Pn3m space group of a spacing parameter equal to 10.4 nm (see S.2.2 of Supplementary Material). This structure is characterized by two sets of interwoven aqueous nanochannels, lined by a lipid bilayer. Direct images of the internal architecture of cubosomes have been collected with Cryo-TEM performed at the FloCEN facility at the University of Florence (Figure 1D and Supplementary Figure S9). The average hydrodynamic diameter of cubosomes (d_h_) was evaluated through Dynamic Light Scattering measurements by analysing the autocorrelation function with a CONTIN Laplace inversion. The obtained cubosomes are characterized by an average diameter peaked at d_h_ = 240 nm and negative ζ-potential (−34 ± 1) (see Supplementary Figures S5, S6 for further details).

### 3.2 AuNPs and AgNPs Interacting With Cubosomes

**FIGURE 2:**
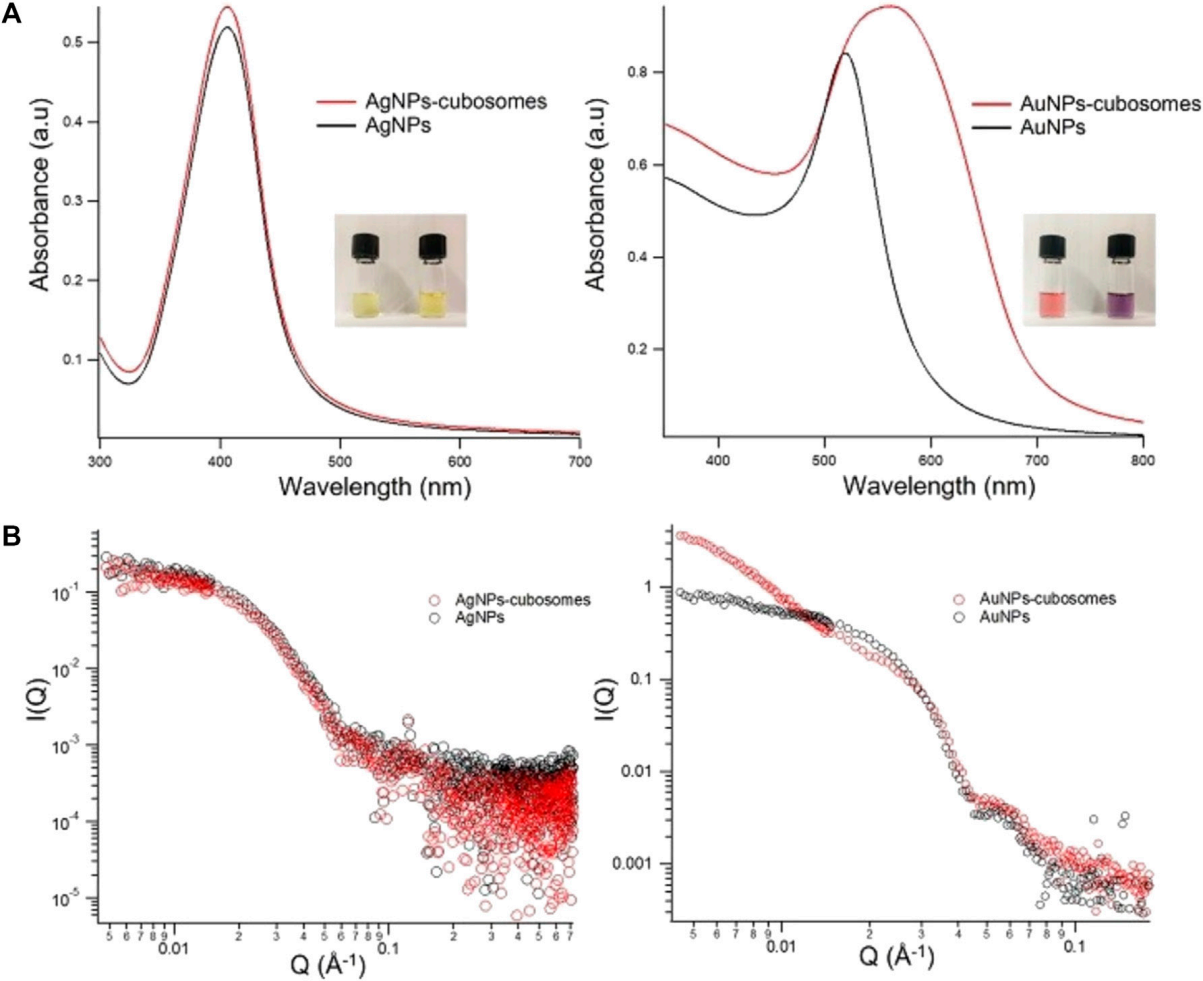
**(A)**: UV-Visible absorption profiles of AgNPs-cubosomes (left) and AuNPs-cubosomes (right). **(B)**: Log-log SAXS profiles of AgNPs-cubosomes (left) and AuNPs-cubosomes (right) hybrids. 300 µL of NPs (8.3 × 10^−10^ M) were mixed with 20 µL of 0.4 mg/ml cubosomes (for a final concentration of cubosomes in the mixture of 0.025 mg/ml) and SAXS and UV-vis profiles were collected after 10 min of incubation. The SAXS profile of cubosomes in water at a 0.025 mg/ml lipid concentration has been subtracted from AgNPs-cubosomes and AuNPs-cubosomes profiles.

Figure 2A shows representative UV-vis spectra obtained by incubating 20 μL of 0.4 mg/ml cubosomes with 300 μL of 0.83 nM Ag or AuNPs dispersions. Since the optical properties of metallic particles are connected to the interparticle distance, the optical variations in the absorbance of metallic NPs can be used to monitor their aggregation. Generally, such aggregation provokes the broadening of the plasmonic peak, the red shift of its maximum, and even the occurrence of a new red-shifted peak depending on the morphology of the aggregates (Cardellini et al., 2022). All these variations, easily monitorable through UV-Vis Spectroscopy, represent an excellent tool to determine the interaction of NPs with their environment in biomimetic studies, colorimetric assays, and biosensing applications, as we recently presented in different studies. (Montis et al., 2014b; Busatto et al., 2018; Montis et al., 2020b; Zendrini et al., 2020; Caselli et al., 2021b). In our case, as displayed from UV-Vis spectra and pictures in Figure 2, the plasmonic features of the AgNPs dispersion remain almost unchanged even after 10 min of incubation with the cubosomes’ dispersion. On the contrary, the colour of the AuNPs dispersion quickly turns from red to purple, associated to a prominent red shift of the maximum of the characteristic plasmonic peak. This is due to the coupling of the surface plasmon modes of proximal NPs, indicating the decrease of the interparticle distance and formation of aggregates. Such a spontaneous aggregation of AuNPs is a concentration-dependent process. The aggregation of the particles gradually increases as the concentration of the cubosomes decreases (see Supplementary Figure S12 for UV-Vis characterization). The higher extent of aggregation, observed at low cubosomes’ amounts, is the clear evidence that the NPs aggregation is a membrane-templated phenomenon that only occurs on the cubosomes’ surface.

To gain structural information on cubosomes/NPs hybrids, we performed SAXS measurements (Figure 2B). It is worth noticing that in our experimental conditions, the scattering signal of cubosomes is negligible, due to the higher electronic density of metallic NPs. Consequently, any variation in the scattering profiles can be exclusively related to variations in the NPs arrangement. In agreement with UV-vis results, the scattering profile of AgNPs is not affected by the presence of cubosomes, indicating a very weak (or absent) interaction. In the case of AuNPs/cubosomes hybrids, the colour change reported in the UV-vis spectra is associated with an increase in the slope of the scattering profile at low-q. The power-law dependence observed in this region, highlighted by a linear trend with positive slope in the I(q) vs q double logarithmic plot, can be related to the formation of NPs fractal aggregates on the cubosomes’ membrane.

Overall, our findings underline dramatic differences in the interaction of NPs with cubosomes, depending on the metallic composition of the particles. Despite the very similar physical and chemical properties (size, morphology, concentration, and surface coating) shared by Ag- and AuNPs, the behavior of the hybrid systems is highly different. Specifically, AuNPs spontaneously cluster on the cubosomes membrane, pointing out that the attractive forces overcome the electrostatic repulsion, while the aggregation is inhibited in the case of AgNPs. However, UV-vis spectroscopy and SAXS measurements only focus on the behaviour of the inorganic NPs, without providing any information on their effect on the cubic membrane.

### 3.3 Cubosomes Deposition on the Lipid Bilayer

The preparation of model lipid membranes in the form of cubosomes adsorbed onto a solid support allowed us to exploit surface techniques (such as QCM and Confocal Microscopy) which allow for monitoring the modifications induced by NPs on the cubic membrane. Such an investigation will complement the information obtained from solution techniques (SAXS and UV-Vis spectroscopy), providing new fundamental insights on the interaction from the membrane perspective. Moreover, it enabled to set the focus exclusively on phenomena occurring at the nano-bio interface (i.e., where metallic NPs meet cubic interfaces), ruling out possible bulk effects.

**FIGURE 3:**
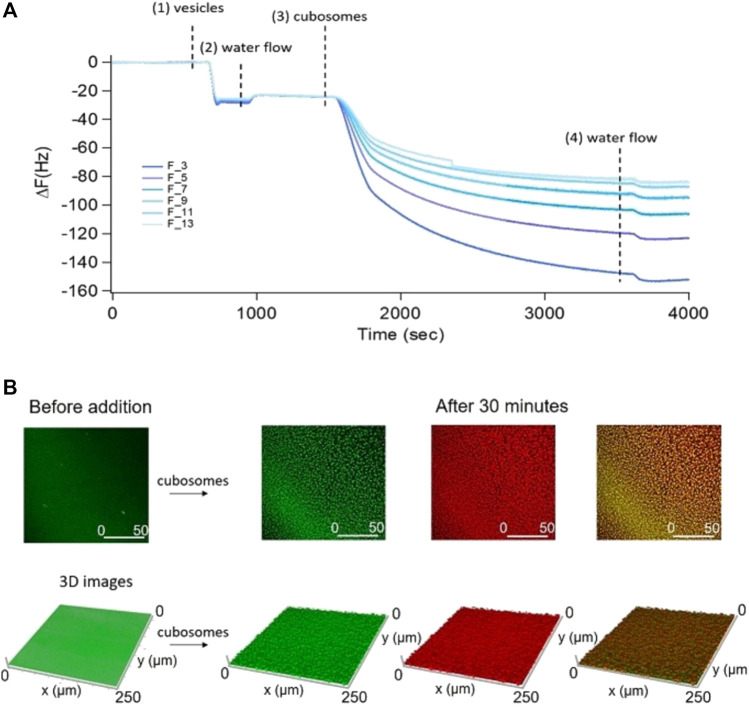
Panel **(A)**: QCM experiment on the adsorption of 4 mg/ml GMO-based cubosomes on a DOPC/DOTAP SLB. Vertical lines represent the subsequential injection of DOPC/DOTAP vesicles, water, cubosomes, and water (1–4). Panel **(B)**: Representative 2D and 3D Confocal Microscopy images of the cubosomes deposition on the SLB. SLB and cubosomes were fluorescently labelled with two different probes with well separate emission spectra. Specifically, DOPC/DOTAP liposomes have been labelled with β-Bodipy (λexc 488 nm, λem 493–614 nm) while stabilizer-free GMO-based cubosomes with 18:1 Cyanine 5 PE (λexc 633 nm, λem 662–732 nm). Cubosomes are imaged in red, the SLB in green and the colocalization of the probes in yellow.

The preparation of solid-supported nanometric assemblies of cubic nature is not straightforward. Most of the studies so far focuses on the interaction of inorganic NPs with model bulk cubic phases (not suitable for surface studies and not well representative of real membranes, characterized by a nanometric thickness). More recently, new methods have been engineered to obtain thinner cubic phase films, with micro (Dabkowska et al., 2017a; Caselli et al., 2022) or nanometric (Ridolfi et al., 2021) thickness. These approaches are based on the spin coating of lipids dissolved in organic solvents (such as chloroform, hexane, and ethanol), generally toxics and complex to get rid of. Here, we exploited a novel approach, which does not require the use of organic solvents. This process is based on the spontaneous deposition and self-assembly of cubosomes onto a solid support, leading to the formation of a thin (i.e. nanometric) cubosomes carpet. Considering that the absorption of GMO-based cubosomes does not take place on bare hydrophilic SiO_2_ substrates due to the negative zeta-potential of these lipid particles, we promoted the adsorption of cubosomes by functionalizing SiO_2_ substrates with a slightly positive Supported Lipid Bilayer (SLB). Considering the negative surface charge of cubosomes, the electrostatic interaction with the positively charged SLB is expected to promote a quantitative coverage of the substrate by cubosomes. We prepared the SLB through the adsorption and fusion on a hydrophilic SiO_2_ substrate of 0.5 mg/ml DOPC (1,2-dioleoyl-sn-glycero-3-phosphocholine)/DOTAP (1,2-dioleoyl-3-trimethylammonium-propane) vesicles (70/30 mol%) dispersed in a 0.1 M NaCl aqueous solution. Figure 3A) shows the formation of the DOPC/DOTAP SLB onto the SiO_2_ support and the subsequent deposition of cubosomes, monitored by QCM. With this technique, the decrease of the resonance frequency (ΔF) can be related to the adsorbed mass on the sensor. First, we formed the SLB by injecting DOPC/DOTAP vesicles in the chamber measurements (step 1) in Figure 3A) with a low flow rate (0.1 ml/min). Once injected into the chamber, the vesicles adhered to the SiO_2_ substrate, provoking a slight decrease of ΔF. Then, the chamber was rinsed with ultrapure water 2) to promote vesicles’ rupture due to the osmotic shock caused by the ionic strength gradient developed across the lipid membrane of vesicles. The inner water content of the vesicles was subsequently released leading to a tiny increase of the ΔF, reaching a stable value of −25 Hz, in perfect agreement with previous reports on SLB formation (Montis et al., 2016). Once the DOPC/DOTAP bilayer was formed, the cubosomes dispersion was injected in the chamber 3). As demonstrated by the high decrease in ΔF, the electrostatic interaction between the lipid bilayer and stabilizer-free cubosomes leads to the instantaneous and massive adsorption of cubosomes. The cell was subsequently rinsed with water (0.5 ml/min flow rate) to check the stability of the cubosomes layer 4); no major variations in ΔF were observed, accounting for the irreversible binding of cubosomes to the SLB, which prevents their detachment under water flow.

**FIGURE 4:**
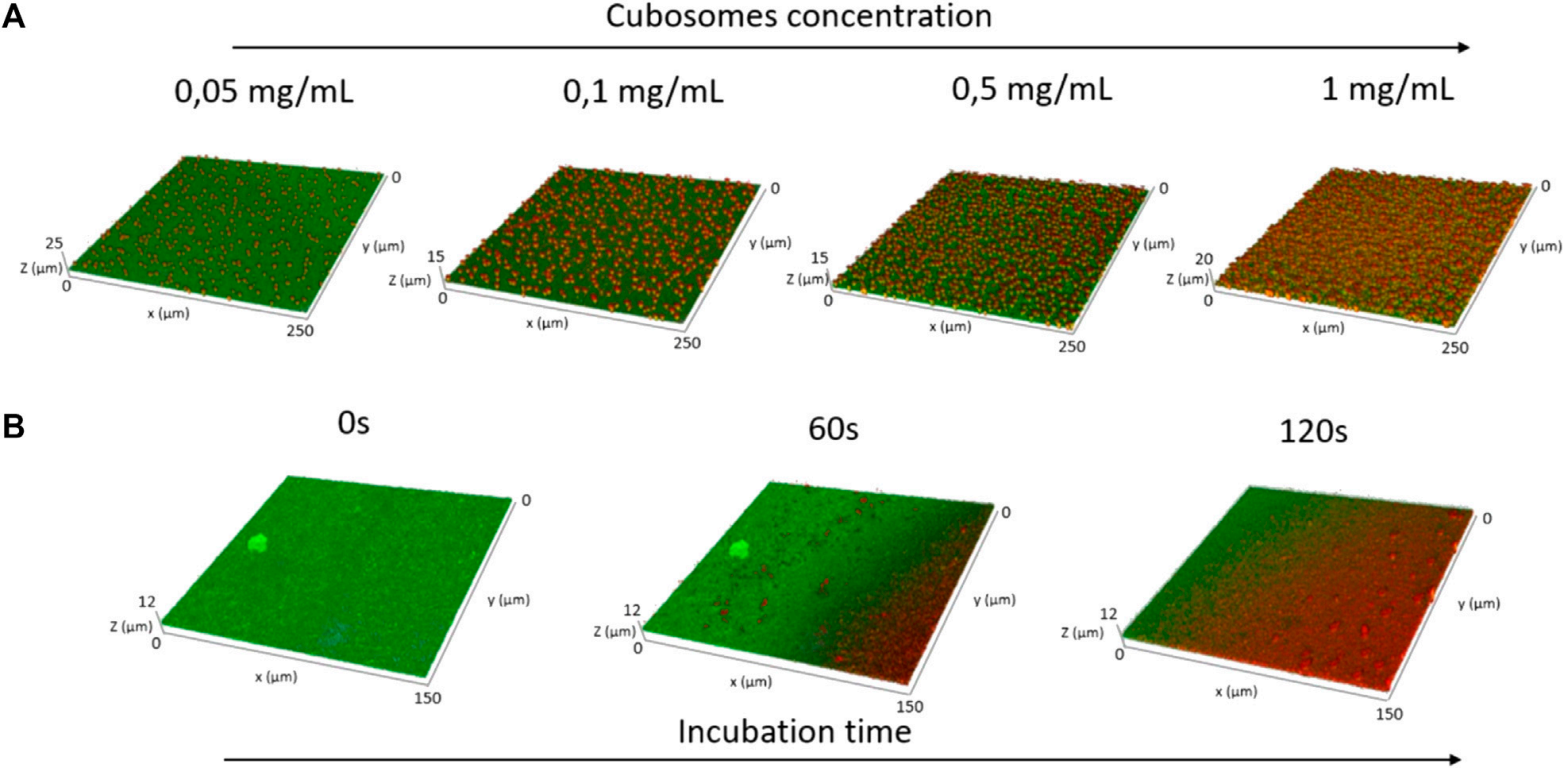
Panel **(A)**: representative CLSM 3D reconstructions of the SLB/cubosomes systems, obtained after 30 min of incubation of the SLB with different concentrations of cubosomes (i.e. 0.05 mg/ml, 0.1 mg/ml, 0.5 mg/ml, and 1 mg/ml). Panel **(B)**: CLSM 3D reconstructions, representing the temporal evolution of the system obtained from the deposition of 0,05 mg/ml cubosomes on the SLB. The 3D reconstructions have been collected at 0s (i.e. before the addition of cubosomes), 60 s, and 120 s from the cubosomes’ addition. DOPC/DOTAP SLB has been labelled with β-Bodipy (λexc 488 nm λem, 493–614 nm) while stabilizer-free GMO-based cubosomes with 18:1 Cyanine 5 PE (λexc 633 nm, λem 662–732 nm). Cubosomes are imaged in red, the SLB in green and the colocalization of the probes in yellow.

As a complementary analysis to directly visualize the formation of the cubosomes layer and obtain structural information at the microscale on coverage, we performed Confocal Laser Scanning Microscopy (CLSM) measurements. For this purpose, we labelled liposomes and cubosomes with two different probes (0.1% mol) with well-separated emission spectra. Representative side and top view 2D images and 3D reconstructions of the SLB before the addition of cubosomes are reported in the Supplementary Material (Supplementary Figure S10). Figure 3B reports representative top view 2D images and 3D reconstructions of the SLB/cubosomes system, collected after 30 min of incubation of 1 mg/ml cubosomes with the lipid bilayer (see Supplementary Figure S11, for corresponding side view 2D images). Here, fluorescence from cubosomes is represented in red, the one from the DOPC/DOTAP SLB in green, while their superposition in yellow. The addition of the cubosomes’ dispersion to the lipid bilayer results in the homogeneous deposition of the cubic particles (red spots) on the SLB (green layer) without membrane disruption. After 30 min, the lipid bilayer is homogeneously covered by a layer of cubosomes. The adsorption kinetic has been also monitored live (see Supplementary Movie S1), showing that only few minutes (<3 min) are required to reach the fully covered state shown in Figure 3B. To gain further insight on the process of deposition of cubosomes, we collected CSLM images of the SLB/cubosomes system, obtained by incubating cubosomes at different concentrations (from 0.05 mg/ml to 1 mg/ml) and monitoring the deposition during the first minutes of interaction (Figures 4A,B4). We found that the deposition of cubosomes is strictly dependent on concentration. Specifically, the pattern of cubosomes on the SLB gets less and less densely packed with decreasing concentrations of cubosomes (Figure 4A). Interestingly, the concentration of cubosomes also affects their dynamic of deposition, which appears much slower for low cubosomes’ amounts (see also Supplementary Movie S2). With a 0.05 mg/ml cubosomes’ concentration, the kinetic of the process is slow enough to allow us to monitor the deposition process in detail, by capturing the morphology of the system under non-equilibrium conditions (i.e, before reaching the final equilibrium forms depicted in Figure 4A). Figure 4B reports representative 3D reconstructions of the mixed SLB/cubosomes (0.05 mg/ml) system at short incubation times (<5 min). In these experimental conditions, we noticed that the (green) SLB assumes a homogeneous red coloration within the first seconds from the addition of cubosomes. This is likely to be due to a fast exchange of material, which includes the fluorescent probe, occurring when cubosomes firstly dock on the SLB substrate. After that, the characteristic red spots start to pattern the support, quickly reaching an equilibrium configuration (within 10 min). The intrinsic resolution limit in CSLM does not allow to unambiguously assess whether such red spots are single -and isolated-cubosomes, or small cubosomes clusters. However, the negative surface charge of cubosomes (see Section 3.1) implies a strong electrostatic attraction with the positively charged surface of the SLB, responsible for their adsorption; on the contrary, the net repulsion between different cubosomes of same charge is likely to prevent their aggregation on the SLB, leading to the formation a pattern of single -and self-avoiding- cubosomes, such as the one observed in Figures 3, 4.

### 3.4 AuNPs and AgNPs Interacting With Thin Films of Cubosomes

**FIGURE 5:**
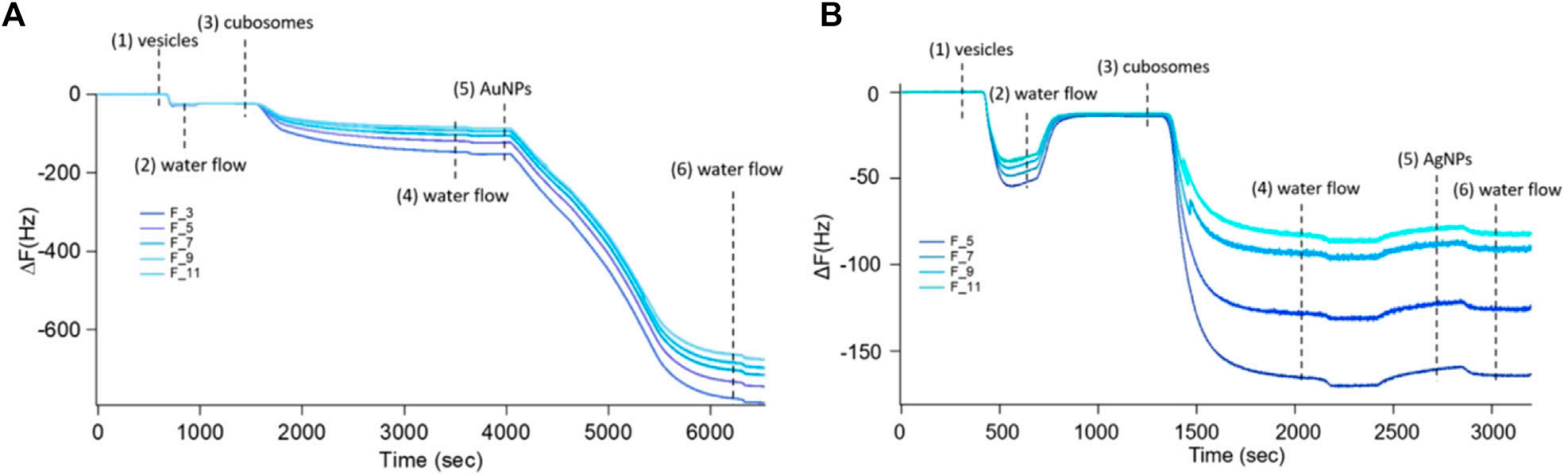
QCM experiments describing the adsorption of 0.83 nM AuNPs (Panel **(A)**) and AgNPs (Panel **(B)**) on the cubosomes film deposited upon the DOPC/DOTAP substrate. The vertical lines represent the subsequential injection of DOPC/DOTAP vesicles (1), milliQ water (2), cubosomes (3), milliQ water (4), AuNPs (left) or AgNPs (right) (5), and milliQ water (6).

Figure 5 displays the NPs adsorption on the cubosomes’ film monitored with QCM. After the formation and stabilization of the thin film of cubosomes (see Section 3.2, steps 1–3), the system was extensively rinsed with milliQ water (step 4) to remove the excess of non-adsorbed cubosomes. Right after, water dispersions of AuNPs or AgNPs (Figure 5B) at a concentration of 0.83 nM were injected into the measuring cell at 0.1 ml/min flow rate (step 5). The resulting interaction perfectly mirrors what observed for the corresponding dispersed systems. AuNPs strongly interact with the substrate, producing an immediate and dramatic decrease in the oscillation frequency (Figure 5A); this indicates an elevated mass adsorption, which starts immediately and reaches its maximum within the first 30 min of interaction. Importantly, flushing milliQ water at a considerable flow rate (i.e. 0.5 ml/min) does not induce any appreciable removal of the adsorbed AuNPs (step 6), which accounts for a significant AuNPs-cubosomes interaction strength. On the contrary, no relevant shifts of the oscillation frequency were detected following the injection of AgNPs in the QCM chamber (Figure 5B), even after 10 min from the injection. Thus, in this latter case, just a negligible (or even absent) number of particles interact with -and adsorb on-the substrate.

On the same systems, we performed Confocal Microscopy measurements to gain information on the morphological modification possibly induced by NPs on the cubosomes film. Such modifications, conveniently monitored at the micron-scale, would account for the impact of NPs on representative portions (50 × 50 µm squared areas) of the cubosomes membrane. With the same set-up employed previously (see Section 3.2), we simultaneously collected the fluorescent signals from the supported DOPC/DOTAP bilayer and the GMO-based cubosomes, to investigate the impact of NPs at different depths of the lipid assembly. Moreover, we observed the interaction in transmission mode as well, to monitor the possible formation of NPs clusters.

**FIGURE 6:**
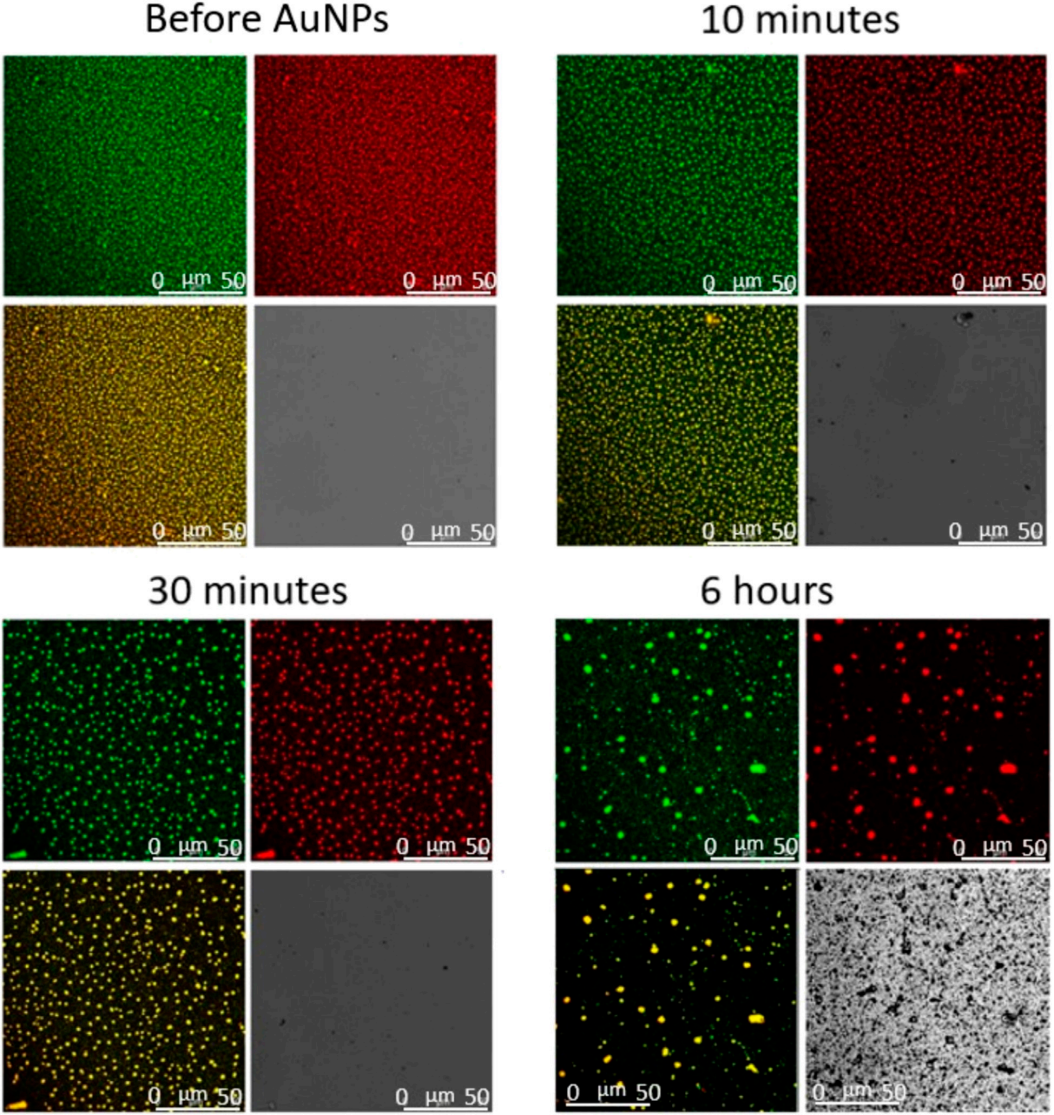
Representative 2D Confocal Microscopy images of the interaction of AuNPs 0.83 nM with the film of cubosomes deposited on the DOPC/DOTAP SLB. Images collected at different incubation times (before and after 10 min, 30 min, and 6 h from AuNPs injection).

Figure 6 shows representative top-view fluorescence and transmission images of AuNPs interacting with the cubosomes layer adsorbed on the DOPC/DOTAP SLB. The time-evolution of the substrate morphology was monitored over a time-period of 6 h. Before injection of the particles, the substrate is characterized by a stable and densely packed cubosomes’ array. Once the particles’ dispersion (0.83 nM) is introduced, the red spots (cubosomes) in the fluorescence image progressively increase in size, hinting to the agglomeration of adsorbed cubosomes. Mirroring the same trend, the SLB below (green fluorescence) becomes less compact. At the same time, the absolute fluorescence intensity of both the lipid bilayer and cubosomes dramatically decreases, suggesting that a lipid extraction process takes place. After 6 h of interaction, only few large aggregates of mixed lipid composition are still present on the substrate, while the cubosomes layer has completely lost its characteristic packing. The loss of compactness occurs through a progressive dewetting-like process, in which pores are formed within the membrane, similarly to what previously was observed for lamellar membranes interacting with biologically relevant molecules, e.g. polyunsaturated ω-3 fatty acids (Angelova et al., 2019); here, the lipid assembly gradually loses adhesion to the glass substrate and retracts into large lipid agglomerates, likely due to the lipid removal induced by AuNPs. In addition, the transmission image shows micron-sized AuNPs (dark spots), which extensively cover the glass surface.

**FIGURE 7:**
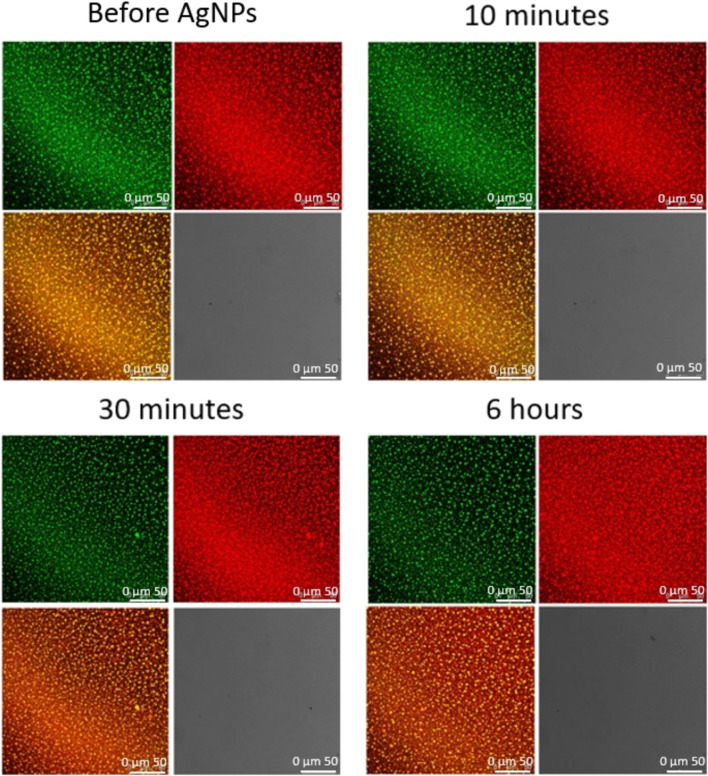
Representative 2D Confocal Microscopy images of the interaction of AgNPs 0.83 nM with the film of cubosomes deposited on the DOPC/DOTAP SLB. Images collected at different incubation times (before and after 10 min, 30 min, and 6 h from AuNPs injection).

The very same experiment was performed incubating the cubosomes carpet with AgNPs at the same concentration (Figure 7). Fully in line with previous measurements, the top-view images show that citrated AgNPs do not affect the morphology of the substrate in the first 6 h of interaction. Moreover, the transmission signal doesn’t present any sign of NPs aggregation in the chosen experimental time.

Overall, confocal microscopy investigation showed that the interaction with the cubosomes layer is strictly dependent on the particle composition. After a relatively long incubation time, AgNPs don’t affect the morphology of the substrate and no micron-sized clusters of particles are detected. On the contrary, the effect of AuNPs is much stronger. AuNPs start to interact with the substrate extracting lipids from both the lipid bilayer and the cubosomes. The homogeneous cubosomes’ pattern becomes less and less densely packed with time. After 6 h of incubation the substrate is almost completely disrupted, concentrated in isolated spots, and partially substituted by extended AuNPs clusters.

## 4 Discussion

In this study we compared for the first time the interaction of a highly curved biomimetic interface of cubic nature with two prototypical metallic nanoparticles for biomedical applications: AuNPs and AgNPs, characterized by a citrate surface coating and a spherical shape. SAXS and UV-vis data, performed in solution, provided information on the variation of the NPs arrangement and interaction at the nanoscale, while Confocal Microscopy gave access to alterations of the micron-sized morphology of the cubic phase supported membrane. Being the selected NPs almost identical in terms of size, concentration and surface coating, our experimental results revealed that the interaction is governed—both at the nano and at the microscale-by the metallic composition of the particles. In particular, we showed that: 1) at the nanoscale: AuNPs quickly cluster on the cubosomes membrane, leading to the formation of NPs’ fractal aggregates. On the contrary, the presence of the cubic phase membrane does not induce appreciable aggregation of AgNPs; 2) at the micron-scale: the ordered array of SLB-supported cubosomes gets readily un-packed and concentrated into separated dewetted-like spots by the action of AuNPs, while it is almost unaffected upon contact with AgNPs.

**FIGURE 8:**
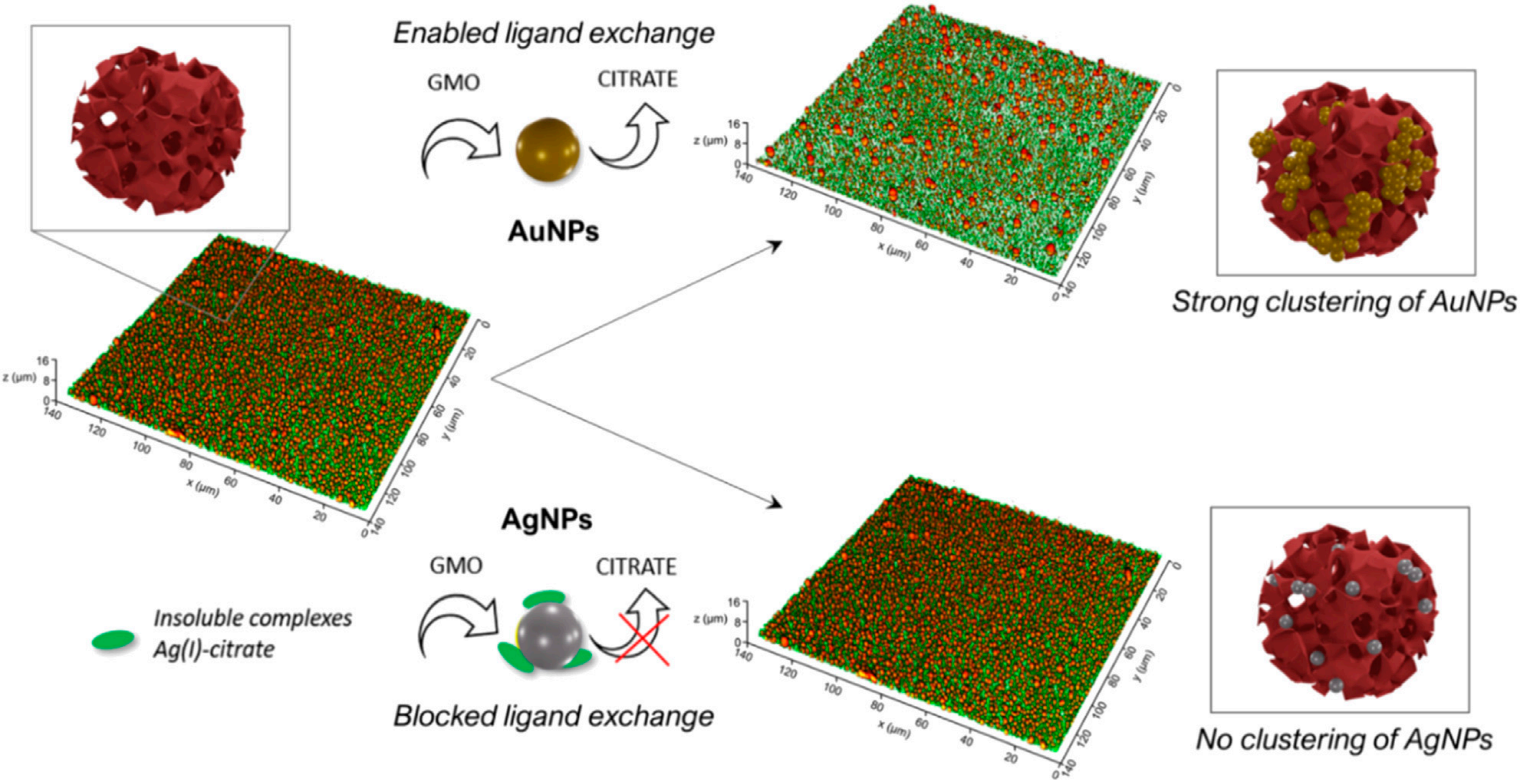
Schematic representation of the mechanism and final outcomes of the interaction of AuNPs and AgNPs with a water dispersion cubosomes and solid-supported films of cubosomes.

In recent works, a combination of computational and experimental results highlighted that the spontaneous aggregation of citrated AuNPs on vesicles of lamellar nature is triggered by the fast release of citrate anions at the AuNPs surface (Montis et al., 2020b; Salassi et al., 2021; Cardellini et al., 2022). Such release occurs upon adhesion of AuNPs to the vesicles’ membrane, which drives the displacement of citrate anions by the lipids composing the vesicles’ membrane (i.e., a citrate/lipid ligand exchange). This causes a loss of electrostatic stabilization of AuNPs, driving their aggregation on the membrane of vesicles. It is reasonable to assume that, in the present case, the aggregation of AuNPs on the cubic membrane occurs through a similar mechanism, i.e., though a ligand exchange between citrate anions, weakly bonded to the gold surface, and the lipids (GMO) constituting the membrane of cubosomes. This ligand exchange, driven by hydrophobic and Van der Waals forces between the gold surface and the lipid molecules, would initiate the AuNPs aggregation on the surface of cubosomes dispersed in aqueous solution (UV-Vis and SAXS data of Figure 2), and provoke the lipid extraction and substrate removal observed on solid-supported cubosomes films (Figure 6). Figure 8 (upper part) schematizes such mechanism of interaction and its final outcomes on both dispersed and solid-supported cubosomes.

From this perspective, the different behavior of AgNPs may be explained considering that citrate molecules cannot be as easily displaced from the silver surface as from the gold one. As reported in several works (Djokić, 2008; Anees Ahmad et al., 2020; William et al., 2021), citrate anions form water-insoluble complexes with Ag(I) ions, originated from solubilised Ag at the silver surface. Such complexes lie on the surface of AgNPs, partially -or completely-covering it, and are not readily displaceable at the lipid/water interface. Thus, once AgNPs are mixed with the cubosomes, the formation of these complexes would hamper the ligand-exchange reaction with lipids, preventing the citrate anions release (see Figure 8, downer part). As a result, no clustering of AgNPs is revealed on dispersed cubosomes (see UV-Vis and SAXS data of Figure 2), and no lipid removal and membrane disruption are observed in solid-supported cubosomes (see also CLSM data of Figure 7).

## 5 Conclusion

In the past years, the interaction of inorganic NPs and biomimetic membranes has been intensively investigated to increase our fundamental knowledge on nano-bio interfaces, develop novel synthetic smart hybrid nanomaterials and to predict their biological fate. However, most of these studies focused on planar lamellar membranes. Only recently, synthetic cubic lipid assemblies have been introduced in the library of biomimetic systems, to provide fundamental insight into the role of non-lamellar interfaces in natural systems, such as cubic membranes. Despite these very recent advancements, the biological role of cubic interfaces and their response to NPs adhesion remains an almost unexplored research field. Here for the first time, we present a physicochemical investigation of the interaction of cubic phase interfaces -both as cubosomes dispersions in water and as thin layers adsorbed on a substrate-with two prototypical NPs particularly relevant for biomedical applications, i.e. AgNPs and AuNPs. The cubosomes’ surface array here introduced, represents a particularly convenient structural platform to test NPs’ behavior towards cubic phase lipid biomimetic interfaces.

Through an ensemble of different techniques, we disentangled the impact of such NPs on cubic phase structures at different length scales, i.e., from the nano-to the micron-scale. As observed for synthetic lamellar systems, citrated AuNPs cluster on the membrane of dispersed cubosomes, and extensively aggregate on a solid-supported cubosomes’ film, leading to its disruption. On the contrary, AgNPs with the same size and identical surface coating, do not affect the morphology of the solid-supported cubosomes films and don’t cluster on dispersed cubosomes. This experimental evidence may be related to the binding energy of the citrate molecules to the different metallic surfaces. Citrate anions are weakly associated to the gold surface and can be easily released though a ligand-exchange with the lipids, promoting the aggregation of AuNPs. Conversely, citrate anions form insoluble complexes with the Ag(I) ions at the silver surface, which stabilizes the colloidal dispersion and hampers the interaction with cubic membranes. These results contribute shedding light on the prominent role of the nature and association between ligands and metallic NPs surfaces in determining the events at nano-bio interfaces of cubic nature.

## Data Availability

The original contributions presented in the study are included in the article/Supplementary Material, further inquiries can be directed to the corresponding author.
