# Supplementary material for: Interaction of Metallic Nanoparticles With Biomimetic Lipid Liquid Crystalline Cubic Interfaces
Source: Front Bioeng Biotechnol. 2022 Mar 15;10:848687. doi: 10.3389/fbioe.2022.848687 (PMC8964527; doi:10.3389/fbioe.2022.848687)
Supplement: Supplementary file 1 [file DataSheet1.pdf]

Supplementary Material

Interaction of Metallic Nanoparticles with Biomimetic Lipid Liquid Crystalline Cubic Interfaces

Jacopo Cardellini[1,2], Costanza Montis[1,2], Francesco Barbero[2,3], Ilaria De Santis,[1,2] Lucrezia Caselli[1,2,4,*] and Debora Berti[1,2]

[1]Department of Chemistry "Ugo Schiff", University of Florence, Via della Lastruccia 3, Sesto Fiorentino, Florence 50019, Italy

[2]CSGI, Consorzio Sistemi a Grande Interfase, University of Florence, Sesto Fiorentino, Italy

[3]Present Address Department of Chemistry, University of Turin, francesco.barbero@unito.it

[4]Department of Pharmacy, University of Copenhagen, DK-2100 Copenhagen, Denmark

*** Correspondence:**
Dr. Lucrezia Caselli
lucrezia.caselli@sund.ku.dk

Keywords: gold nanoparticles, silver nanoparticles, biomimetic systems, cubic phases, cubosomes, nano-bio interface, lipid liquid crystals, nano-bio interactions.

	Index	*Page*
S.1	***Supplementary Characterization of Gold and Silver Nanoparticles***	***3***
**S.1.1**	Small Angle X-ray Scattering	3
**S.1.2**	UV-Vis Spectroscopy: AuNPs	5
**S.1.3**	UV-Vis Spectroscopy: AgNPs	6
S.2	***Supplementary Characterization of Cubosomes***	***7***
S.2.1	Dynamic Light Scattering and Zeta-Potential	7
S.2.2	Small Angle X-ray Scattering	8
S.2.3	Cryo-TEM	9
S.3	***Supplementary Characterization of cubic thin films***	***9***

S.3.1	DOPC/DOTAP Supported Lipid Bilayer formation	9
S.4	***Supplementary Characterization of AuNPs-cubosomes hybrids***	***10***
**S.4.1**	UV-vis Spectroscopy: Effect of cubosomes concentration	10
S.5	***Bibliography***	***11***

S.1 Supplementary Characterization of Gold and Silver Nanoparticles

S1.1 Small Angle X-ray Scattering

SAXS measurements on AuNPs aqueous dispersion were carried out in sealed glass capillaries of 1,5 mm diameter.

In diluted solution without interparticle interaction, the structure factor S(Q) can be approximated equal to 1, and the scattering intensity I(q) assumes the following form:

$$I(Q)=n\Delta\rho^2 V_p^2 P(Q) \qquad (1)$$

Where n is the number density of the objects in the dispersion, $\Delta\rho$ is the contrast between the solvent and the scattering objects, V_p is the particle's volume and P(Q) is the form factor. Within the Guinier approximation[1], valid for diluted and monodispersed particles, P(Q) can be expressed as:

$$P(Q) = 1 - \frac{Q^2 R_g^2}{3} \qquad (2)$$

And substituting equation (1) we can obtained:

$$I(Q) = n\Delta\rho^2 V_p^2 \exp\left(- \frac{Q^2 R_g^2}{3}\right) \qquad (3)$$

And in logarithmic form:

$$\ln I(Q) = \text{cost} - \frac{Q^2 R_g^2}{3} \qquad (4)$$

Then, according to the Guinier approximation, when S(Q)=1, the slope of scattering profile in the low Q region in a ln I(Q) vs Q^2 plot can be associated to the average gyration radius of the particles if the equation $QR_g<1$ is respected. Finally, we can extract the particle's radius, assuming a spherical shape, exploiting the following relation:

$$R = \sqrt{\frac{5}{3}}\, R_g \qquad (5)$$

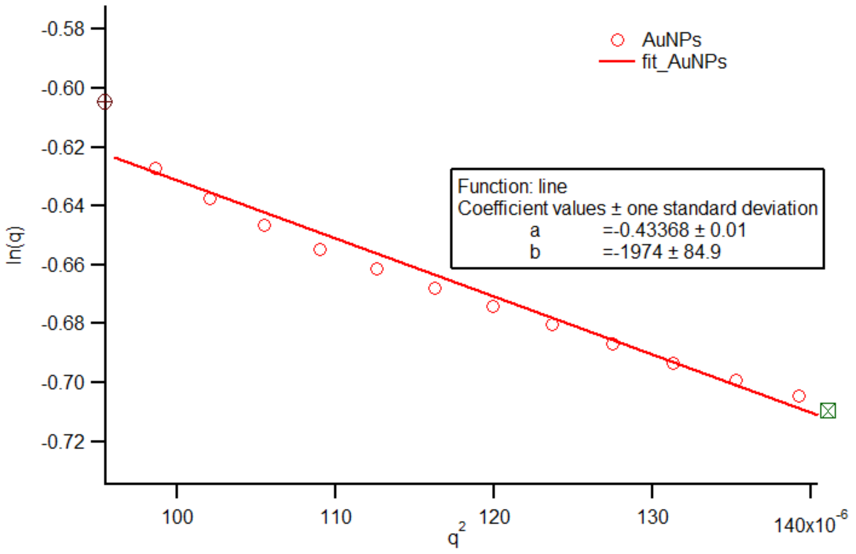

Figure S1 *Guinier approximation for gold nanoparticles: ln I(q) vs q^2 plot. The slope of linear fitting (solid red line) of the scattering intensity in the Guinier Region (red markers) is related to the gyration radius of the particles. The size and polydispersity obtained from the fitting procedure are summarized in the Table S1 below.*

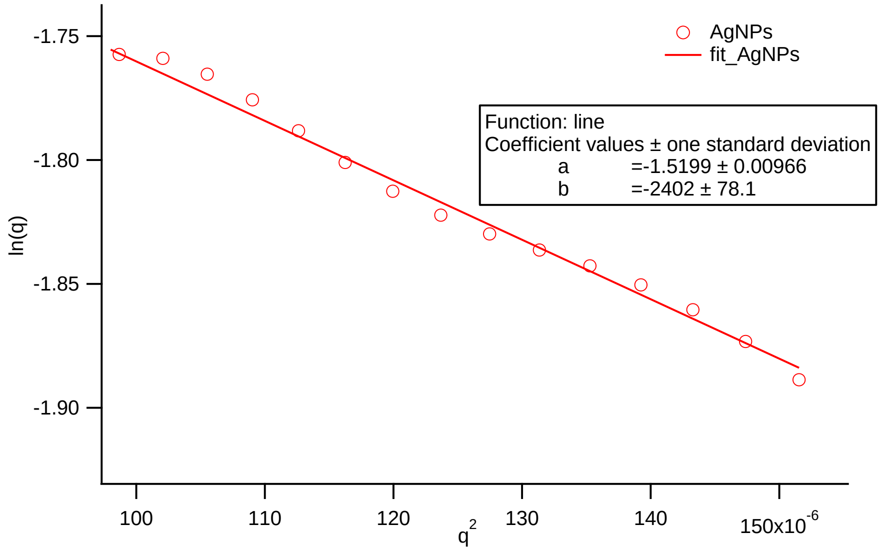

Figure S2 *Guinier approximation for silver nanoparticles: ln I(q) vs q^2 plot. The slope of linear fitting (solid red line) of the scattering intensity in the Guinier Region (red markers) is related to the gyration radius of the particles. The size and polydispersity obtained from the fitting procedure are summarized in the Table S1 below.*

	R_{core} (nm)
**AuNPs**	20 ± 1
**AgNPs**	22 ± 1

Table S1 *Nanoparticles radii obtained for AuNPs and AgNPs for the analysis of the SAXS profiles according to the Guinier approximation.*

UV-vis Spectroscopy: AuNPs

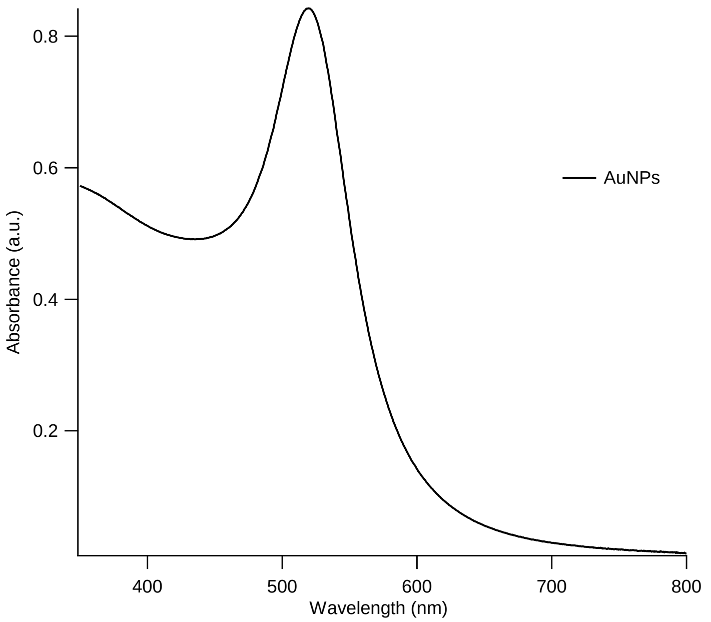

Figure S3 *UV-Vis spectra of AuNPs after 1:3 dilution in water (2.77x10^{-10}M). The plasmon absorption peak is at around 520 nm.*

To further evaluate the AuNPs size through UV-Vis spectroscopy we exploited the following equation[2]:

$$d = \exp\left(B_1 \frac{A_{spr}}{A_{450}} - B_2\right) \qquad (6)$$

with d diameter of gold nanoparticles, A_{spr} absorbance at the surface plasma resonance peak, A_{450} absorbance at the wavelength of 450 nm and B_1 and B_2 are dimensionless parameters, taken as 3 and 2,2, respectively. The diameter value obtained is of 20 nm.

The concentration of citrated gold nanoparticles was determined via UV-Vis spectrometry, using the Lambert-Beer law ($E(\lambda) = \varepsilon(\lambda)lc$), taking the extinction values $E(\lambda)$ at the LSPR maximum, i.e. $\lambda = 520$ nm. The extinction coefficient $\varepsilon(\lambda)$ of gold nanoparticles dispersion was determined by the method reported in literature[3], by the following equation:

$$\ln(\varepsilon) = k\ln(d) + a \qquad (7)$$

with d core diameter of nanoparticles, and k and a dimensionless parameters ($k = 3{,}32111$ and $a = 10{,}80505$). The arithmetic mean of the sizes obtained by optical and scattering analyses was selected, leading to a $\varepsilon(\lambda)$ of $2.0 \cdot 10^{8}$ $M^{-1}cm^{-1}$. The final concentration of the citrated AuNPs is therefore $\sim 8.3 \cdot 10^{-10}$ M.

UV-vis Spectroscopy: AgNPs

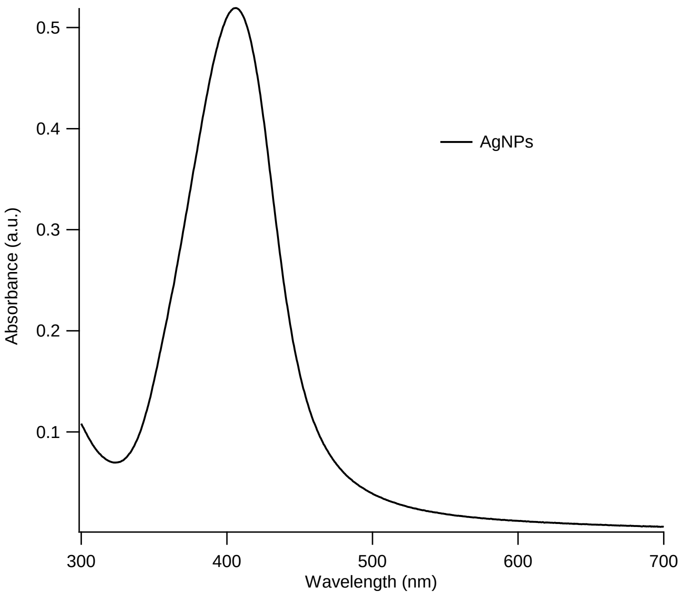

Figure S4 *UV-Vis absorption spectra of AgNPs after 1:3 dilution in water ($1.6x10^{-9}M$). The plasmon absorption peak is at 520 nm.*

To confirm the AgNPs size evaluated by SAXS we exploited the UV-vis spectroscopy according the following equation[4]:

$$d = 0.11\ (\lambda_{max})^2 - 89.99\ (\lambda_{max}) + 17{,}775.94 \qquad (8)$$

where d is the diameter and λ_{max} is the wavelength corresponding to the maximum absorption peak. Exploiting this equation, we found a nanoparticle's diameter equal to d=18.8 nm.

The concentration of citrated AgNPs was determined via UV-Vis spectrometry, using the Lambert-Beer law ($E(\lambda) = \varepsilon(\lambda)lc$). The extinction coefficient $\varepsilon(\lambda)$ of silver nanoparticles dispersion was determined by the method reported in literature, according to the following equation:

$$\varepsilon = 0.202e^{0.251d} \quad (9)$$

The final concentration obtained for AgNPs is $1.8x10^{-8}$ M. Finally, before each measurement, the AgNPs dispersion was diluted to the same concentration of AuNPs ($8.3x10^{-10}$ M).

Supplementary Characterization of Cubosomes

Dynamic Light Scattering and Zeta-Potential

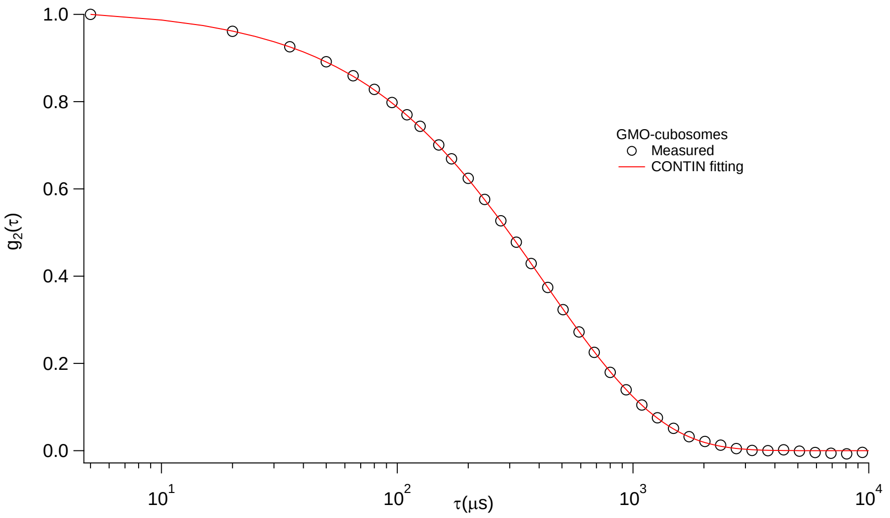

Figure S5 *Dynamic Light Scattering curve of GMO-cubosomes dispersion analyzed with a Contin through the Laplace inversion according to the CONTIN algorithm*[5].

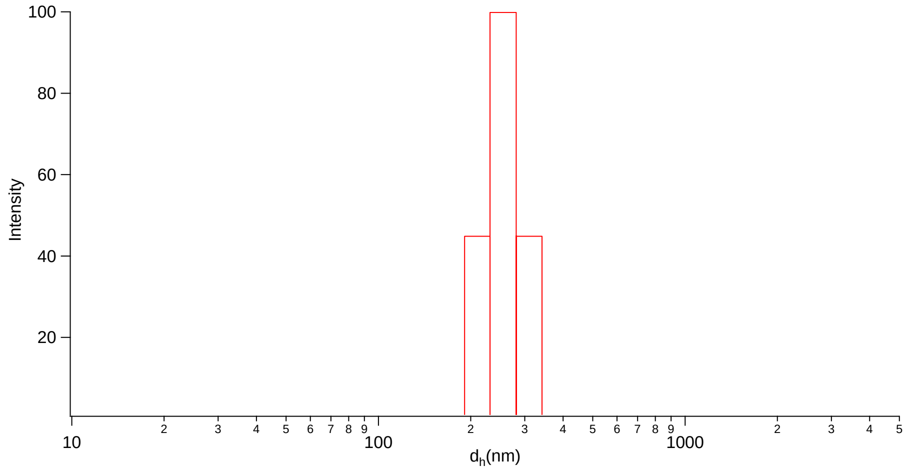

Figure S6 *Size distribution of cubosomes obtained by the Contin analysis.*

Small Angle X-Ray Scattering

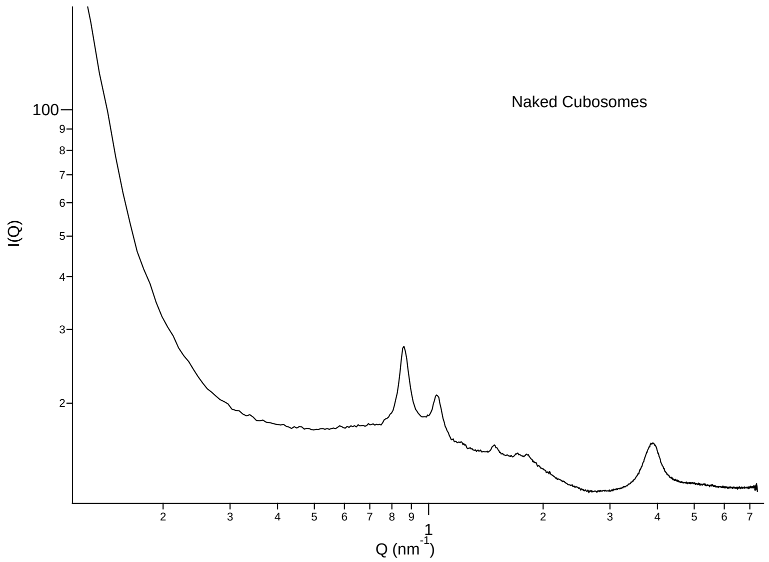

Figure S7 *Comparison between the scattering intensity of water and the scattering intensity of 10 µL of liposome dispersions (12 nM) in 300 µL of water.*

In order to obtain the spacing parameter of the Pn3m cubic phase we exploit the following equation[6]:

$$Q_{hkl} = \frac{2\pi}{d} * (h^2 + k^2 + l^2)^{1/2} \qquad (10)$$

Where h, k and l are the Miller indexes representing the crystallographic planes of the liquid crystallin phase and Q_{hkl} is the q position measured for each reflex. In the case of a Pn3m phase, the characteristic values of $(h^2 + k^2 + l^2)^{1/2}$ are $\sqrt{2}$, $\sqrt{3}, \sqrt{4}, \sqrt{6}, \sqrt{8}$ and $\sqrt{9}$. Thus, plotting x=$Qhkl$(1/Å) vs y=(h2+k2+l2)1/2, the spacing parameter d can be obtained by the slope b of the liner fitting, reported in figure S7, according to the following equation:

$$d = \frac{2\pi}{b} \qquad (11)$$

The evaluated spacing parameter is 10.4 nm.

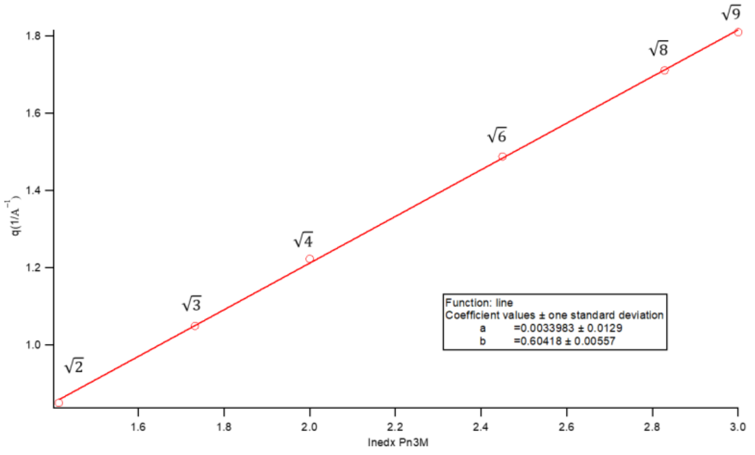

Figure S8 *Comparison between the scattering intensity of water and the scattering intensity of 10 µL of liposome dispersions (12 nM) in 300 µL of water.*

Cryo-TEM

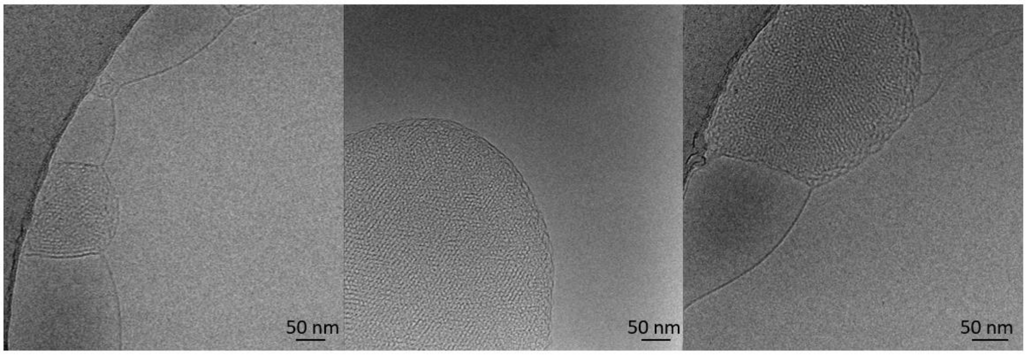

Figure S9 *Further examples of cryo-TEM images of stabilizer-free GMO cubosomes.*

Supplementary Characterization of cubic thin films

DOPC/DOTAP Supported Lipid Bilayer formation

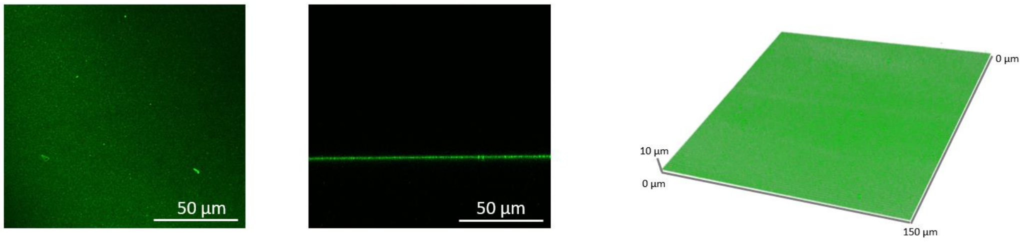

Figure S10 *Top view (left), side view (middle) and 3D view (right) of DOPC/DOTAP supported lipid bilayer collected with Confocal Microscopy.*

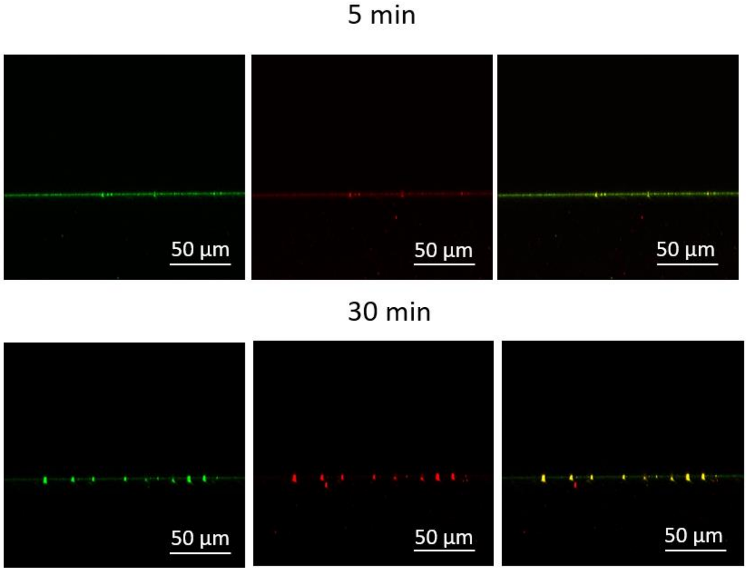

Figure S11 *Side views of the cubosomes deposition on the DOPC/DOTAP SLB 5 min (top) and 30 min (bottom) after the cubosomes injection. Cubosomes are imaged in red, the SLB in green and the colocalization of the probes in yellow.*

Supplementary Characterization of AuNPs-cubosomes hybrids

UV-vis Spectroscopy: Effect of cubosomes concentration

We monitored the AuNPs aggregation varying the cubosomes concentration. 300 µL of AuNPs $8.3x10^{-10}$ M were incubated with 0, 15, 30, 60, and 120 µL of cubosomes 4 mg/mL and the optical variations of the dispersion were evaluated after 10 minutes of interaction. Figure S12 displays the collected UV-visible spectra. As shown, increasing the number of cubosomes, the optical variation of the original AuNPs spectrum become less pronounced. This experimental evidence underlines that the AuNPs clustering is templated by the cubosomes, and the particles aggregation occurs on the membrane. Moreover, it is maximized by decreasing the available surface for aggregation until the saturation of this surface is reached. These findings are totally in agreement with the self-assembly of citrated AuNPs on synthetic vesicles composed of zwitterionic phospholipid, extensively studied in a recent work.[7]

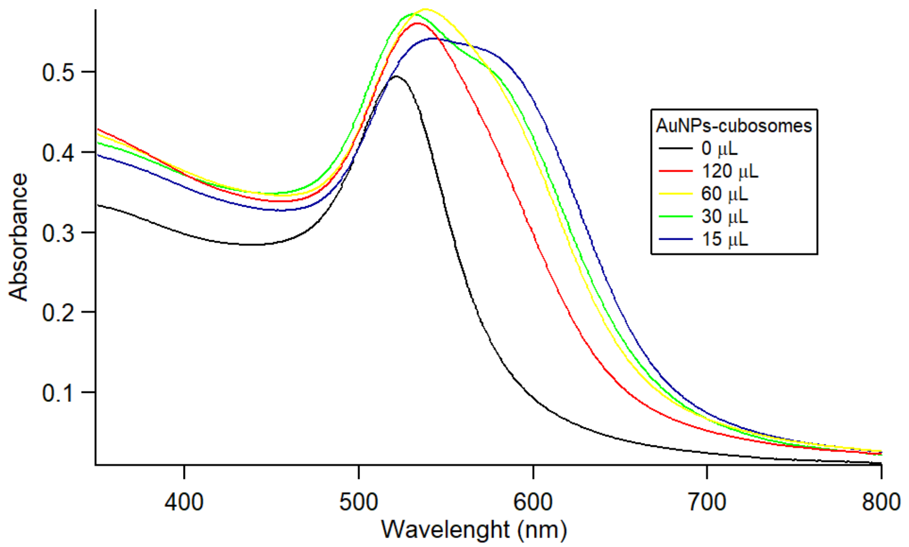

Figure S12 *UV–Vis spectra of AuNP (0.83 M) in the presence of different amounts of GMO-based cubosomes (120 µL, 60 µL, 30 µL, 15 µL and 0 µL of 4 mg/mL cubosomes).*

Bibliography

1 H. B. Stuhrmann, *Prog. Cryst. Growth Charact.*, 1989, **18**, 1–19.

2 W. Haiss, N. T. K. Thanh, J. Aveyard and D. G. Fernig, 2007, **79**, 4215–4221.

3 X. Liu, M. Atwater, J. Wang and Q. Huo, 2007, **58**, 3–7.

4 M. Sikder, J. R. Lead, G. T. Chandler and M. Baalousha, *Sci. Total Environ.*, 2018, **618**, 597–607.

5 S. W. Provencher, *Comput. Phys. Commun.*, 1982, 27, 229–242.

6 C. A. F. Peters and J. Kortazzi, *Astron. Nachrichten*, 1873, **82**, 145–148.

7 C. Montis, L. Caselli, F. Valle, A. Zendrini, F. Carlà, R. Schweins, M. Maccarini, P. Bergese and D. Berti, *J. Colloid Interface Sci.*, 2020, **573**, 204–214.
